# Slower upper extremity function in older adults with hyperkyphosis negatively impacts the 6-min walk test

**DOI:** 10.1186/s12891-022-05455-x

**Published:** 2022-05-28

**Authors:** Yoshimi Fukuoka, Wendy B. Katzman, Amy Gladin, Nancy E. Lane, Deborah M. Kado, Yoo Jung Oh

**Affiliations:** 1 Department of Physiological Nursing, University of California, San Francisco, 2 Koret Way, N631, San Francisco, CA 94143 USA; 2 Department of Physical Therapy and Rehabilitation Science, University of California, San Francisco, San Francisco, CA 94158 USA; 3 San Francisco Medical Center, Kaiser Permanente Northern California, San Francisco, CA 94118 USA; 4 Center for Musculoskeletal Health, School of Medicine, University of California at Davis, Davis, CA 95616 USA; 5 Department of Medicine, Stanford University School of Medicine, Stanford, CA and Geriatric Research Education and Clinical Center (GRECC), Veterans Health Administration, Palo Alto, CA 94304 USA; 6 Department of Communication, University of California Davis, Davis, CA 95616 USA

**Keywords:** Older adults, Kyphosis, Cobb angle, 6-min walk test, Upper extremity task, Physical function, Hyperkyphosis

## Abstract

**Background:**

Approximately 30% to 40% of older adults have hyperkyphosis, defined as excessive curvature of the thoracic spine. Hyperkyphosis is associated with increased morbidity and mortality. This study aimed to determine whether hyperkyphosis (Cobb’s angle) and upper extremity tasks were independently associated with the 6-min walk test (6MWT) in community-dwelling older adults with hyperkyphosis.

**Methods:**

In this cross-sectional study, we studied 71 women and 28 men aged 60–87 from the study of hyperkyphosis, exercise, and function trial (SHEAF) who had kyphosis, 3 timed upper extremity tasks and the 6MWT assessed at their baseline visit. We used standing lateral spine radiographs and a standardized protocol for thoracic kyphosis (T4-T12) to measure Cobb angle of kyphosis. In addition, 3 activity of daily living (ADL) extremity tests (putting on and removing a laboratory coat, picking up a penny from the floor, and lifting a 7-lb. book to a shelf) were used.

**Results:**

The mean ± SD age was 70.1 ± 6.1 years. The mean ± SD Cobb angle of kyphosis was 57.4 ± 12.5 degrees. On average ± SD, the participants walked 504.8 ± 84.2 m in 6 min and took 2.4 ± 2.2 prescription medications. The mean ± SD height was 164.7 ± 8.5 cm, weight was 68.7 ± 13.1 kg, and BMI was 25.2 ± 4.0 kg/m^2^. Multivariate regression revealed that age, height, upper extremity book lift task, and the number of prescribed medications were significant predictors of performance on the 6MWT (*p* < 0.05).

**Conclusions:**

While kyphosis was not associated with the 6MWT, timed tests of upper extremity function indicated that upper body dynamics can affect walking performance. In addition, sociodemographic factors and the number of prescribed medications were significant contributing factors to the 6MWT in older adults with mild to moderate hyperkyphosis. These results illustrate multifactorial influences on physical performance and the need for an integrated and targeted approach in helping older hyperkyphotic adults maintain healthy physical functioning as they age.

## Background

Hyperkyphosis, or stooped posture, is a common and easily recognizable condition among older adults, and it progresses with age. Hyperkyphosis is defined as excessive thoracic spine curvature, and the gold-standard diagnostic test is a radiographic Cobb angle greater than 40 degrees. Approximately 30% to 40% of older adults have hyperkyphosis [1, 2]. Hyperkyphosis is associated with impaired physical function and increased morbidity and mortality [3]. In particular, hyperkyphosis with 54 degrees or greater substantially increases fall risk. Given a rapidly growing older adult population [4], we expect that the prevalence of hyperkyphosis will increase. Developing a more comprehensive understanding of the physical and functional correlates in older adults with hyperkyphosis will help elucidate the problem and provide the rationale for its prevention and treatment.

The 6-min walk test (6MWT) is a simple assessment to measure how an individual can walk on a flat, hard surface in 6 min [5]. This test involves all the bodily systems for walking, particularly musculoskeletal, cardiovascular, and respiratory systems. Good test–retest reliability, and convergent and construct validity of the 6MWT were previously reported to measure functional exercise capacity and physical endurance in older adults [6–8]. Several systematic reviews of factors influencing the 6MWT distance were conducted in adults with cardiovascular disease, chronic respiratory disease, acute respiratory distress syndrome, community-dwelling adults, and children/adolescents [9–13]. Despite the popularity of the 6MWT, there are limited data relating to the 6MWT in older adults with hyperkyphosis.

Furthermore, trunk and upper extremity strength and range of motion can influence walking abilities, and severe hyperkyphosis may impair upper extremity function. However, upper extremity functions in relation to the 6MWT distance have not been carefully examined in community-dwelling older adults in general and, in particular, those with hyperkyphosis. A previous study reported that upper extremity functions showed significant correlations with the 6-min walk distance (6MWD) in middle-aged to older patients with chronic obstructive pulmonary disease (COPD) [14]. However, to the best of our knowledge, the association between upper extremity function and 6MWT has not been examined in older adults with hyperkyphosis. Therefore, the aim of this study was to determine if hyperkyphosis and upper extremity tasks were independently associated with the 6MWD in community-dwelling older adults with hyperkyphosis.

## Methods

### Study design, sample, and procedures

In this cross-sectional, secondary data analysis study, we analyzed 99 older adults who participated in the baseline visit for the study of hyperkyphosis, exercise, and function (SHEAF) randomized controlled trial (RCT). The study protocol was approved by the University of California San Francisco and Kaiser Permanente Northern California Institutional Review Boards, and the Data Safety Monitoring Board members before participant enrollment. Detailed descriptions of the study design and protocol and participant eligibility have been previously published [15, 16]. In brief, participants were recruited from local senior centers, outpatient medical clinics, physician referrals, and medical center databases in San Francisco, California.

Inclusion criteria were proficiency in English, age ≥ 60 years, kyphosis angle ≥ 40° by the Debrunner kyphometer (Techmedica Inc, Camarillo, California) measured at the screening visit, and ability to walk one block without the use of an assistive device, climb one flight of stairs independently, and rise from a chair without the use of one’s arms. Exclusion criteria were inability to straighten the thoracic spine at least 5°, cognitive impairment (unable to draw a normal clock or recall any words on the Mini-Cog) [17, 18], inability to pass safety tests in the screening examination, or any disorder or disease likely to prevent or interfere with safe participation in a group-based exercise class (see methods paper for details on safety tests, disorders, and diseases) [15]. In total, 598 participants were pre-screened for eligibility. Of those, 290 were excluded for not meeting pre-screening criteria or for being disinterested in the study. The remaining 308 proceeded to clinic screening. However, 209 participants were excluded due to not meeting eligibility criteria or declining to participate in the study, resulting in 99 participants who ultimately enrolled. We obtained permission from the participant’s primary care provider and all participants provided written informed consent. All baseline data collections were conducted before the randomization.

### Measures

#### 6MWT 

The 6MWT measured the distance in meters covered while walking along a standardized-distance of 60-feet in a long hallway marked by two cones for 6 min. This test measures the distance that a participant can walk on a flat, hard surface in 6 min [19]. It evaluates the global and integrated responses of systems involved during exercise, including the pulmonary and cardiovascular, circulatory, neuromuscular, and musculoskeletal. A previous study has concluded the 6MWT to be more reflective of activities of daily living than other walk tests [20]. In the original trial, all participants except one could complete the 6MWT at baseline.

#### Average baseline steps per day

We used an Omron pedometer to objectively measure the baseline physical activity level for 7 consecutive days before the randomization visit.

#### Cobb angle of kyphosis

It was measured using the gold standard Cobb angle of kyphosis derived from standing lateral spine radiographs and a standardized protocol for thoracic kyphosis (T4-T12) [21]. Participants stood barefoot with knees straight and arms supported at 90° of shoulder flexion; they were instructed to hold full inhalation for the duration of the scan. A trained radiologist (BF) made measurements to read the radiographs. A greater Cobb angle indicates more kyphosis severity. Test–retest reliability for repeated measurement of Cobb angle from the same radiograph was estimated as ICC = 0.90. The standard error of the measurement was estimated as 1.4° [16].

#### Kyphosis from kyphometer

The Debrunner kyphometer (Techmedica Inc., Camarillo, CA) was used for external measurement of kyphosis using the T2/3 spinous process interspace as the superior landmark and T11/T12 spinous process interspace as the inferior landmark.

#### Upper extremity function

In this study, 3 activity of daily living (ADL) extremity tests (putting on and removing a laboratory coat, picking up a penny from the floor, and lifting a 7-lb. book to a shelf) were used. These were 3 components of the modified Physical Performance Test (modified PPT) [22, 23], a 9-item standardized test, including 7 timed and two untimed upper and lower extremity ADL tasks, used to assess multiple domains of physical function. The timed-book lift test and timed-putting on and removing a laboratory coat are upper extremity ADL tasks, and the timed pick-up a penny from floor task is a combined upper and lower extremity ADL task. Participants completed all 9-items in the modified Physical Performance Test.

#### Weight, height, and body mass index (BMI)

Individuals with hyperkyphosis could have two height measures (usual and best posture height). We measured the usual height in centimeters in a barefoot position in this study. We assessed weight in kilograms with light clothing and calculated BMI as weight in kilograms divided by height in meters squared.

**Vertebral fractures** were assessed in T4 to L4 vertebrae by an experienced radiologist from the baseline standing lateral spine radiographs using the Genant semiquantitative (SQ) method grading fractures 0–3 [24].

#### Demographic and medication

Demographic information, such as age, sex, race/ethnicity, education, were collected from participants at the baseline visit. In addition, trained research staff asked participants to report the names of any medication/supplements they were regularly taking. The number and types of prescribed and non-prescribed medications were recorded, and prescribed medications were categorized into blood pressure, hyperlipidemia, hypothyroidism, diabetes, anxiety/depression, arthritis, and osteoporosis medications.

### Statistical analysis

Descriptive statistics were used to describe participants’ sociodemographic (age, gender, race, education) and clinical information (height, weight, BMI, 6MWT, Cobb’s angle, kyphosis, vertebral fractures, baseline steps, 3 extremity ADL tasks, number and types of medication). To investigate the association between sociodemographic and clinical factors on participants’ performance on the 6MWT (m), univariate and multivariate regression analyses were conducted. The multivariate regression model tested whether baseline Cobb’s angle and 3 extremity ADL measures were independently associated with the 6MWT performance after controlling for sociodemographic and clinical characteristics. A sensitivity analysis was conducted by replacing Cobb’s angle with the Debrunner kyphosis reading in the multivariate regression model. Statistical significance was set at *P* < 0.05. All analyses were conducted using SPSS (version 21.0; IBM, Chicago, IL, USA).

## Results

### Sample characteristics

Table 1 shows the sample sociodemographic and clinical characteristics at baseline. Of the 99 participants, the mean ± SD age was 70.1 ± 6.1 years with a range from 60 to 87 years, 71 (71.7%) were women, 86 (86.9%) were white, 78 (78.8%) completed college or graduate school. Mean ± SD height was 164.7 ± 8.5 cm, weight was 68.7 ± 13.1 kg, and BMI was 25.2 ± 4.0 kg/m^2^. On the 6MWT, participants walked 504.8 ± 84.2 m. Mean ± SD Cobb’s angle was 57.4 ± 12.5 degrees and kyphosis was 54.1 ± 8.6 degrees. A total of 16 (16.2%) participants reported two or more vertebral fractures. The average ± SD baseline steps were 5691.3 ± 3186.4 per day. Regarding three ADL tasks (i.e., book lift, jacket, pick up penny tasks), the average ± SD seconds taken to complete the task was 2.7 ± 0.7 s for the book lift task, 8.7 ± 2.6 s for jacket task, and 1.5 ± 0.4 s for pick up penny task. Our results for ADL tasks were similar to findings on older adults of similar age and BMI (2.7 s for book lift, 6.6 s for jacket, and 2.2 for pick up penny task [25]). Lastly, the mean ± SD number of prescription medications was 2.4 ± 2.2.

**Table 1:** Sample sociodemographic and clinical data at baseline (*N* = 99)

Sociodemographics		Mean (SD) or %(n) [Range]
Age (years)	**-**	70.1 (6.1) [60–87]
Gender	Men	28.3 (28)
	Women	71.7 (71)
Race/Ethnicity	White	86.9 (86)
	Non-white	13.1 (13)
Education	Less than high school, completed high school, or some college education	21.2 (21)
	Completed college or graduate school	78.8 (78)
**Clinical data**		
Height (cm)	-	164.7 (8.5) [145–184]
Weight (kg)	-	68.7 (13.1) [44–114]
Body Mass Index (kg/m^2^)	-	25.2 (4.0) [17.8–39.7]
Number of medication/supplement	-	5.3 (3.5) [0–15]
Number of prescription medications	-	2.4 (2.2) [0.0 – 11.0]
Taking blood pressure medication	Yes	34.3 (34)
Taking hyperlipidemia medication	Yes	32.3 (32)
Taking hypothyroidism medication	Yes	15.2 (15)
Taking diabetes medication	Yes	4.0 (4)
Taking anxiety/depression medication	Yes	10.1 (10)
Taking arthritis medication	Yes	6.1 (6)
Taking medication(s) for osteoporosis Alendronate. Other antiresorptive medication, parathyroid hormone, or other born-building medications	Yes	16.2 (16)
6-minute walk test (m)^a^	-	504.8 (84.2) [293.7–804.0]
Cobb’s angle (**°)**^b^	-	57.4 (12.5) [29.1 – 89.2]
Kyphosis	-	54.1 (8.6) [35–73]
Vertebral fractures (Y)^c^	Yes (2 or more)	16 (16.2)
Average baseline steps (per day)^d^	-	5691.3 (3186.4) [943–17558]
Book lift task (seconds)	Yes / seconds	98.0 (97) / 2.7 (0.7) [1.2–5.6]
	No	2.0 (2)
Jacket task (seconds)	Yes / seconds	100 (99) / 8.7 (2.6) [3.7–17.0]
	No	0 (0)
Pick up penny task (seconds)	Yes / seconds	100 (99) / 1.5 (0.4) [0.7–3.1]
	No	0 (0)

^a^1 missing case

^b^5 missing cases

^c^2 missing cases

^d^26 missing cases

### Regression analyses

Table 2 presents the results of univariate regression analyses and a multivariate linear regression analysis predicting individuals’ performance on the 6MWT at baseline. Overall, the multivariate regression model was significant (adjusted R^2^ = 0.382, *p* < 0.001). Specifically, it revealed 4 significant predictors: (1) age (B = -3.37; 95% CI, -5.89 to -0.85; *p* = 0.009), (2) height (cm) (B = 2.76; 95% CI, 0.30 to 5.22; *p* = 0.029), (3) Book lift seconds (B = -37.58; 95% CI, -63.80 to -11.36; *p* = 0.006), and (4) number of prescribed medication taken (B = -8.17; 95% CI, -15.03 to -1.31; *p* = 0.020). This indicates that individuals with younger age and taller height were significantly more likely to perform better in the 6MWT than their counterparts. Furthermore, older adults who took less time completing the book lift task and took less prescribed medications showed better performance in the 6MWT. The sensitivity analysis result showed that kyphosis when replacing Cobb’s angle with Debrunner angle in the multivariate regression analysis did not change the results.

**Table 2:** Unadjusted and adjusted regression on the 6-minute walk test at baseline (*N* = 91)

	**Unadjusted**				**Adjusted**^**a**^			
	**B**	**t**	***p*-value**	**95% CI**	**B**	**t**	***p*-value**	**95% CI**
Age	-2.85	-2.03	.045	-5.63 to -0.06	-3.37	-2.66	.009	-5.89 to -0.85
Female gender	-38.02	-2.05	.043	-74.77 to -1.26	-15.91	-0.72	.474	-59.89 to 28.07
Non-white	-58.12	-2.37	.020	-106.73 to -9.50	-36.69	-1.65	.104	-81.06 to 7.68
Completed college or graduate school	24.32	1.18	.243	-16.74 to 65.37	31.25	1.74	.085	-4.44 to 66.94
Height (cm)	3.44	3.65	< .001	1.57 to 5.31	2.76	2.23	.029	0.30 to 5.22
Weight (kg)	0.01	0.02	.987	-1.29 to 1.31	-1.29	-1.88	.063	-2.65 to 0.07
Vertebral fractures	26.36	1.12	.264	-20.24 to 72.96	-15.29	-0.72	.471	-57.32 to 26.75
Cobb’s angle (**°)**	0.70	1.01	.316	-0.68 to 2.09	0.08	0.14	.887	-1.09 to 1.25
Book lift seconds	-52.15	-4.81	< .001	-73.71 to -30.60	-37.58	-2.85	.006	-63.80 to -11.36
Jacket seconds	-6.89	-2.13	.036	-13.31 to -0.47	-0.45	-0.14	.891	-6.96 to 6.06
Penny seconds	-59.83	-3.09	.003	-98.27 to -21.40	-13.58	-0.64	.525	-55.91 to 28.75
Number of prescribed medication	-12.28	-3.28	.001	-19.71 to -4.85	-8.17	-2.37	.020	-15.03 to -1.31

^a^Model R^2^ = .464, Adjusted R.^2^ = .382, *p* < .001

## Discussion

Age-related hyperkyphosis has been defined as a new geriatric syndrome associated with advanced age and increases the risk of physical frailty [1]. However, hyperkyphosis has been underinvestigated because of lack of standardized diagnostic criteria and treatments. To the best of our knowledge, this was the first study to examine whether kyphosis angle degree and upper extremity ADL functions were independently associated with the 6MWT distance in community-dwelling older adults with age-related hyperkyphosis. We believe that the study findings will provide new insights to design health promotion and prevention programs in older adults with hyperkyphosis.

In univariate analyses, all 3 ADL extremity tasks, book-lift, jacket, and pick-up penny off the floor, were associated with 6MWT. This is consistent with prior 6MWT construct validity studies among older adults that report moderate correlation with a broad measure of ADL function (physical function subscale of the SF 36 including both upper and lower extremity function) [7, 26, 27], suggesting 6MWT is broadly associated with ADL function. However, only the book-lift task remained significant after adjusting for socioeconomic and clinical characteristics, suggesting unique body functions independent of other known confounding variables contributing to 6MWT performance. The book-lift upper extremity functional task requires unique body functions including dynamic trunk, scapulothoracic and upper extremity mobility and stability and grip strength to lift the 7-pound book to the shelf compared to the other two ADL tasks, which require upper extremity motion and coordination, balance, and lower extremity squatting ability. A cross-sectional study of older women with hyperkyphosis, osteoporosis, and vertebral fractures, found moderate correlations between a trunk and upper extremity endurance and strength test and 6MWT and ADL function, but not hyperkyphosis, suggesting unique contributions from the trunk and upper extremity strength and endurance [28]. Shoulder range of motion was not measured in the current study and differences in motion could also contribute to performance on book-lift test. The book-lift task requires participants to grip and hold a 7-pound book. Grip strength is a known predictor of frailty [29] and decline in function [30, 31] and was reproted to have moderate correlations with 6MWT in a cohort of Chinese older adults [32]. Supporting the theory that dynamic trunk, scapulothoracic and upper extremity mobility and endurance contribute to the book-lift task performance was a clinical trial that targeted yoga-based trunk and upper extremity strength and motion for 6-months among a cohort of older adults with hyperkyphosis. The study found that post-intervention kyphosis, book-lift timed-task, shoulder elevation (vertical reach) and dynamic (during normal and fast gait) scapular stability and static scapular positioning improved. However, in contrast to trunk and scapular strength and endurance, also contributing to 6MWT, gait-speed and chair stand time did not improve (lower extremity function), which require similar body structure functions for the 6MWT [33, 34]. Furthermore, while the parent clinical trial study did evaluate gait-speed, chair stands time and Timed Up and Go, all of which require similar body functions to the 6MWT, we did not include these variables in the regression analysis as previous investigators have reported moderate to excellent criterion validity of these measures with the 6MWT in mixed populations (older adults, stroke and partial spinal cord)[35, 36]. Further study is needed to understand the relationships between the trunk and upper extremity strength and endurance, physical function, and hyperkyphosis in older adults with hyperkyphosis.

To the best of our knowledge, no studies have investigated the association between the 6MWT and Cobb angle of kyphosis in older adults with age-related hyperkyphosis. While we hypothesized that a greater degree of kyphosis would negatively impact the 6MWT, we did not find an association between the Cobb angle of kyphosis and the 6MWT. The inconsistencies could be explained by the multifactorial nature of the Cobb angle of kyphosis. First, our cohort included high functioning community-dwelling adults with age-matched normative values for the 6MWT [37]. They were an active group with a mean daily step count of 5691.3 (3186.4) steps, greater than the 4400 steps per day found to reduce the risk of mortality [38]. Second, kyphosis is known to progress slowly in adults over 50 years of age, approximately 3 degrees each decade of life [39], and adaptations were likely made to maintain exercise capacity on the 6MWT. Third, we excluded participants with a fixed kyphosis and those without at least 5 degrees of mobility in the thoracic spine (to facilitate change from the exercise intervention); thus, this reduced possible restrictions in pulmonary function from kyphosis that could impair the 6MWT [40]. Thus, while age-related hyperkyphosis may increase the risk for physical frailty and impaired physical function, other factors, including physical activity level, may significantly affect physical function more than the degree of Cobb angle curvature alone.

In this study, fewer prescription medications, younger age, and taller height were significant predictors for better 6MWT performance. The number of prescription medications to the 6MWT performance is intuitive. Older adults with more prescription medications probably had more chronic illnesses. The study participants reported using fewer prescription medications than what might be considered average for their age group [41]. Age and height findings were consistent with previous systematic reviews of the 6MWT in various populations [9, 10, 12, 13]. In particular, Salbach found that age, height, weight, and heart rate explained the largest variance of the 6MWT performance [9]. Similar findings were reported in adults with chronic respiratory disease [10] and healthy adult and adolescent samples [12, 13]. Age and height were related to individuals’ walking speed and stride length [42], which may have factored into their 6MWT performance. Some systematic reviews reported that lower weight was associated with better 6MWT performance. However, weight was not a significant predictor, and the study sample could explain this finding with relatively low weight in the present study. These findings highlight the importance of adjusting age and height when interpreting the 6MWT results.

### Strengths and limitations

The strength of this paper includes the use of the gold standard kyphosis measure, Cobb angle of kyphosis derived from standing radiographs, and measuring objective upper extremity function measures. However, despite these strengths, several limitations also need to be acknowledged. The sample in the present study did not include older adults with severe hyperkyphosis or multiple comorbidities who were not able to participate in an exercise and posture training program. Also, the doses of prescribed medication were not available, so the severity of the diseases was not able to be ascertained. In addition, the sample size was relatively small and represented highly motivated subjects who agreed to participate in a 6-month training program. Thus, the study results may not be generalizable to older adults with severe hyperkyphosis or multiple comorbidities. Furthermore, the study sample included predominantly white, college educated, and high functioning older persons, reflecting an important limitation towards generalizability. Finally, this was a cross-sectional observational study of a randomized clinical trial with likely unmeasured confounding. Thus, causal relationships cannot be inferred.

## Conclusions

This study suggests that older adults who experience difficulty performing upper extremity book lift tasks and who are taking multiple prescription medications were less likely to perform well on the 6MWT. Given the expected increase in the prevalence of hyperkyphosis in the aging population, this study suggests the need for health care providers to assess the risk of impairments in upper extremity function and 6MWT in older adults with hyperkyphosis. Lastly, the study’s findings will need to be cross-validated with larger studies that include diverse samples (e.g., ethnicity) to examine whether the effects of one’s age, height, extremity ADL tasks, and a number of prescription medications would be similar across individuals with diverse sociodemographic backgrounds.

## Data Availability

The datasets used in this paper are available from the corresponding author on reasonable request.

## Abbreviations

ADL: Activity of daily living
BMI: Body mass index
6MWT: 6-Minute walk test

## Declarations

### Statement on institutional/national/international guidelines followed for the study

We followed the guidelines on Strengthening the Reporting of Observational Studies in Epidemiology (STROBE).
